# Long Noncoding RNA NONHSAT079852.2 Contributes to GBM Recurrence by Functioning as a ceRNA for has-mir-10401-3p to Facilitate HSPA1A Upregulation

**DOI:** 10.3389/fonc.2021.636632

**Published:** 2021-07-08

**Authors:** Ningning Zhao, Jiajie Zhang, Lili Zhao, Xiaoni Fu, Qian Zhao, Min Chao, Haiyan Cao, Yang Jiao, Yaqin Hu, Chao Chen, Liang Wang, Huijuan Wang

**Affiliations:** ^1^ College of Life Sciences, Northwest University, Xian, China; ^2^ Department of Neurosurgery, Tangdu Hospital of Air Force Medical University, Xian, China

**Keywords:** recurrent glioblastoma multiforme, RNA-sequencing, lncRNAs, HSPA1A, ceRNA

## Abstract

Glioblastoma multiforme (GBM) is the most common brain malignancy and major cause of high mortality in patients with GBM, and its high recurrence rate is its most prominent feature. However, the pathobiological mechanisms involved in recurrent GBM remain largely unknown. Here, whole-transcriptome sequencing (RNA-sequencing, RNA-Seq) was used in characterizing the expression profile of recurrent GBM, and the aim was to identify crucial biomarkers that contribute to GBM relapse. Differentially expressed RNAs in three recurrent GBM tissues compared with three primary GBM tissues were identified through RNA-Seq. The function and mechanism of a candidate long noncoding RNA (lncRNA) in the progression and recurrence of GBM were elucidated by performing comprehensive bioinformatics analyses, such as functional enrichment analysis, protein–protein interaction prediction, and lncRNA–miRNA–mRNA regulatory network construction, and a series of *in vitro* assays. As the most significantly upregulated gene identified in recurrent GBM, HSPA1A is mainly related to antigen presentation and the MAPK signaling pathway, as indicated by functional enrichment analysis. HSPA1A was predicted as the target gene of the lncRNA NONHSAT079852.2. qRT-PCR revealed that NONHSAT079852.2 was significantly elevated in recurrent GBM relative to that in primary GBM, and high NONHSAT079852.2 expression was associated with the poor overall survival rates of patients with GBM. The knockdown of NONHSAT079852.2 successfully induced tumor cell apoptosis, inhibited the proliferation, migration, invasion and the expression level of HSPA1A in glioma cells. NONHSAT079852.2 was identified to be a sponge for hsa-miR-10401-3p through luciferase reporter assay. Moreover, HSPA1A was targeted and regulated by hsa-miR-10401-3p. Collectively, the results suggested that NONHSAT079852.2 acts as a sponge of hsa-mir-10401-3p and thereby enhances HSPA1A expression, promotes tumor cell proliferation and invasion, and leads to the progression and recurrence of GBM. This study will provide new insight into the regulatory mechanisms of NONHSAT079852.2-mediated competing endogenous RNA in the pathogenesis of recurrent GBM and evidence of the potential of lncRNAs as diagnostic biomarkers or potential therapeutic targets.

## Introduction

Glioblastoma multiforme (GBM) is one of the most common and most aggressive primary adult brain tumors. Despite the availability of aggressive treatments, including surgical tumor removal and radiochemotherapy, GBM has a high recurrence rate, and the median survival time of patients with GBM is only 15 months (1–3). Recurrent tumors are less sensitive to chemotherapy than original tumors, and in most cases, a second surgical resection cannot be performed due to invaded functional brain areas. Currently, most research efforts are focused on exploring the pathogenesis of primary GBM, and few studies have emphasized the biology of recurrent GBM (4, 5). Understanding the mechanism of GBM recurrence is of great significance to the treatment of GBM.

Long noncoding RNAs (lncRNAs) are non-protein coding transcripts that are longer than 200 nucleotides in length and regulate gene expression during biological and pathological processes at the epigenetic, transcription, and post-transcription level (6, 7). Substantial evidence indicates lncRNAs play critical roles in tumor initiation and malignant progression. In particular, recent studies reported that lncRNA can interact with miRNAs as a competing endogenous RNA (ceRNA) to participate in expression regulation of target genes. This newly presented model for gene expression regulation may aid to identification of new targets for tumor treatment (8–10).

Recently, a number of lncRNAs have been implicated in the oncogenesis of gliomas and associated with cell proliferation and apoptosis of glioma and the prognosis of GBM patients. Some of them are increasingly being considered potential therapeutic targets (11, 12). Chen et al. reported that NEAT1 can promote GBM cell growth and invasion *via* the WNT/b-catenin pathway (1). Liu et al. confirmed that the lncRNA HOTAIR promotes glioma progression by acting as a competing endogenous RNA for miR-126-5p (13). Another study revealed that the expression level of the lncRNA SPRY4-IT1 in human glioma tissues and cell lines is upregulated and SPRY4-IT1 can suppress cell growth and metastasis; thus, SPRY4-IT1 may be used as a therapeutic target (14). Collectively, these lncRNAs are of great value as novel biomarkers to clinical applications. However, most current studies were performed using primary GBM clinical samples or cells, and the biological roles and functions of lncRNAs in recurrent GBM have not been fully explored.

Therefore, in this study, whole-transcriptome sequencing (RNA-sequencing, RNA-Seq) of recurrent and primary GBM specimens and subsequent comprehensive bioinformatics analyses were performed for the identification of key lncRNAs associated with glioma. Then, the functional characterization of the selected lncRNA in GBM pathogenesis was performed through a series of *in vitro* biological assays.

## Materials and Methods

### Sample Preparation

Six fresh tumor specimens from three cases of primary GBM (two females and one male, sample serial numbers: 01, 02, and 03) and three cases of recurrent GBM (two females and one male, sample serial numbers: 04, 05, and 06) were obtained from the Department of Neurosurgery in Tangdu Hospital of Air Force Medical University. The patients with primary GBM were treatment-naïve before surgery and the patients with recurrent GBM had been treated with temozolomide plus radiotherapy before relapse. The resected specimens were histologically examined using hematoxylin and eosin (H&E) staining (**Supplementary Figure 1**). The patients and/or their family members understood the process of this study, and each patient signed an informed consent. This study was approved by the Medical Ethics Committee of Tangdu Hospital (TDLL-2017-172).

### RNA Extraction, Library Construction, and RNA-Seq

Total RNA was extracted using TRIzol reagent in accordance with the manufacturer’s instructions, then the concentration and purity of the RNA were measured with a NanoDrop ND-1000 spectrophotometer (NanoDrop Technologies, Wilmington, DE, USA). cDNA libraries were constructed using rRNA-depleted total RNAs as templates according to the protocol of the mRNA-Seq sample preparation kit (Illumina, San Diego, USA). The resulting libraries were sequenced on an Illumina Hiseq 2500 platform (Illumina San Diego CA, USA). More than 200 million paired-end reads were generated. We obtained three biological replicates to minimize experimental errors and the number of false positives.

### Differential Expressed Genes

Differentially expressed genes (DEGs) in recurrent GBMs compared with primary GBMs were analyzed using the R software DEGseq (15). A fold change (FC) of ≥2 and false discovery rate (FDR) of <0.05 were considered as the criteria for DEG selection. According to the obtained DEGs, ggplot2 in R software was used in creating a volcano plot and heatmap for drawing DEGs.

### Gene Function Annotation

The differentially expressed mRNAs (DEmRNAs) were functionally annotated through Gene Ontology (GO) and the Kyoto Encyclopedia of Genes and Genomes (KEGG) pathway enrichment analysis, which were performed using the R language version 3.4.4 cluster Profiler. A *P* value of <0.05 was used as the threshold value.

### Protein–Protein Interaction Analysis

According to the DEG results and protein–protein interactions included in the STRING database (https://string-db.org/), protein–protein (PPI) pairs between DEmRNAs were obtained, and Cytoscape (Version 3.7.2) was used in visualizing the PPI network.

### LncRNAs Target Gene Analysis

For a lncRNAs with a known gene symbol, the gene symbol was used in searching information related to target genes in a software database (starBase, http://starbase.sysu.edu.cn/index.php; ChIPBase, http://rna.sysu.edu.cn/chipbase/; and nonecode, http://www.noncode.org/). The correlation between the gene expression levels of differentially expressed lncRNAs (DElncRNAs) and DEmRNAs was evaluated using the Pearson correlation method. mRNAs with absolute value of correlation coefficients of >0.9 and *P* of <0.01 were considered potential targets of lncRNAs.

### miRNA Target Gene Prediction

On the basis of miRNA and human gene sequence information, miRNAs targeting DEmRNAs were predicted using miRanda (http://www.mirbase.orgmiRBase) and targetscan (http://www.targetscan.org/), and miRNA–mRNA regulatory networks were obtained.

### Analysis of the NONHSAT079852.2-Mediated Regulatory Network

LncRNA–miRNA and miRNA–mRNA relationships were established, and lncRNAs and mRNAs that had at least five co-bind miRNAs were defined as competitive RNAs, and ceRNA was established with Cytoscape.

### Histopathological Examinations

For H&E staining, fresh GBM tissues were fixed in 4% paraformaldehyde for 24 h. Paraffin blocks were embedded and then cut into sections. The paraffin sections were stained with H&E. The rabbit anti-human HSPA1A (dilution fold; 1:100; cat. no. A12948) and CPS1 (dilution fold; 1:100; cat. no. 4214) antibodies were purchased from ABclonal Biotech Co., Ltd (Cambridge, USA).

### Cell Lines and Reagents

A human U251 cell line with short tandem repeat (STR) analysis-based identification certificate was purchased from Wuhan Punosai Life Science and Technology Co., Ltd. GBM-W, a primary GBM cell line derived from a clinical GBM specimen, was obtained from Tangdu Hospital and identified through STR analysis (**Supplementary Figure 2**). The cells were cultured in DMEM (Wuhan Punosai Life Technology Co., Ltd.) supplemented with 10% fetal bovine serum and 1% penicillin. The shRNA plasmid used to knock down the expression of lncRNA NONHSAT079852.2 was purchased from Shanghai Jikai Gene Co., Ltd.

### Cell Transfection

The U251 and GBM-W cells were divided into the control (without treatment), shGFP (transfected with a negative control plasmid against GFP), and shRNA (transfected with a shRNA plasmid against NONHSAT079852.2) groups. The plasmids were purchased from Shanghai Jikai Biotechnology Co., LTD. (Shanghai, China). Lipofectamine 2000 (Invitrogen Inc., Carlsbad, CA) was used for cell transfection according to the manufacturer’s instructions. The shRNA targeting sequences for NONHSAT079852.2 were listed in **Supplementary Table 1**. Of three shRNA targeting sequences, shRNA-652 was validated for the most efficient interference of NONHSAT079852.2 by qRT-PCR and WB, and chosen for further study.

### qRT-PCR Validation

Total RNA from the six GBM tissues and cells was isolated using a TRIzol reagent (Invitrogen, USA), which was reverse-transcribed to cDNA with EasyScript^®^ All-in-One First-Strand cDNA Synthesis SuperMix for qPCR (TransGen Biotech, Beijing, China). The expression levels of selected mRNAs (HSPA1A, CPS1, CCL18, CCL8, and CCL5) and lncRNAs (MSTRG.224498.5, MSTRG.65777.2, and MSTRG.150858.14) were determined through qPCR analysis with a Vii7 QRT-PCR system (Thermo Fisher Scientific). Detailed primer sequences were shown in **Supplementary Table 2**.

### Fluorescence *In Situ* Hybridization (RNA FISH) for NONHSAT079852.2

The subcellular location of the NONHSAT079852.2 was determined using an RNA fluorescence *in situ* hybridization kit (Shanghai GenePharma Co., Ltd.) according to the manufacturer’s instructions. The GBM-W cells were washed with PBS and fixed in 4% paraformaldehyde for 15 min. A biotin-labeled probe was coupled with CY3 fluorescent dye was used, and the cells were incubated at 37°C for 37 min. Hybridization was performed at 37°C for 16 h, the slide was washed, and nuclei were stained with DAPI for 15 min. The images were examined through confocal microscopy with original magnification of 1200× (Leica TCS SP5; Leica Microsystems GmbH, Wetzlar, Germany). The probe sequences were listed in **Supplementary Table 3**.

### Western Blot Analysis

Proteins were extracted from the U251 and GBM-W cells, and protein concentration was determined through the BCA method (Pierce, Rockford, IL). Approximately 40 µg of protein was separated using SDS-containing polyacrylamide gels and transferred onto a polyvinylidene fluoride membrane (Millipore, Billerica, MA, USA). A nonspecific antibody was incubated with 5% milk blocking solution for 2 h and then with primary antibody against HSPA1A (cat. no. A12948; ABclonal Biotech; 1:1000) overnight at 4°C. The membranes were washed with TBST buffer and incubated with peroxidase-labeled secondary antibodies (cat. no. SA00001-1; Proteintech) for 2 h at room temperature. The membranes were rinsed with TBST buffer. Protein bands were exposed with an ECL luminescence solution and detected with a chemiluminescence apparatus (Bio-Rad Hercules, CA, USA).

### CCK-8 and Colony-Formation Assays

The U251 or GBM-W Cells at 5×10^4^ cells/well were seeded in 96-well plates. Cell proliferation was determined through Cell Counting Kit-8 assay (Dojindo Laboratories, Kumamoto, Japan) before transfection and 24, 48, and 72 h after transfection. For cell colony formation assay, cells at 2000 cells/cm^2^ were seeded in six-well plates. After 2 weeks, cell colonies were stained using crystal violet and counted.

### Cell Migration and Invasion Assays

The ratio between the matrix glue and basic medium was set at 1:4, and 50 μl of the mixture was added to each upper chamber. After 3 h, 50 μl of the medium containing 1% serum was added to the upper chamber to hydrate the basement membrane, and 100 μl of cell suspension containing 5×10^4^ U251 or GBM-W cells were added. Exactly 400 μl of medium containing 10% serum was added to the lower chamber. After 36 h, the medium was discarded, the substrate glue in the upper chamber was erased, and the cells on the lower surface of the upper chamber were fixed with 4% paraformaldehyde. The cells were washed with PBS and stained with 0.2% crystal violet. The number of migratory/invaded cells from three different fields was determined through microscopy.

### Wound Healing Assay

The U251 and GBM-W cells were seeded in six-well plates and transfected for 24 h. After the cells reached 80% confluence, a 0.5-mm-wide straight scratch was made on a monolayer of the subconfluent cells with a 200 μl sterile pipette-tip. Cell movement during wound closure was recorded through photography with a phase-contrast inverted microscope at three random fields and time points of 0, 24, and 48 h. Migration rate was calculated as follows: migration rate (%)=(original width−closure width)/original width×100%.

### Flow Cytometry Analysis of Cell Cycle and Apoptosis

After the cells were transfected for 48 h, the cells were collected from the flow tube, and cell cycle (BD-Pharmingen Annexin V PE) and apoptosis (BD Pharmingen™ 7-AAD) were detected through flow cytometry (BD Biosciences, USA).

### Luciferase Reporter Gene Assay

The binding sites of NONHSAT079852.2 and hsa-miR-10401-3p were predicted using the miRanda database, and wild-type NONHSAT079852.2-WT containing a binding site and NONHSAT079852.2-MUT luciferase plasmid containing a binding site mutation were constructed. The luciferase plasmids NONHSAT079852.2-WT and NONHSAT079852.2-MUT were co-transfected with hsa-miR-10401-3p mimics into U251 and GBM-W cells. After 48 h, the luciferase activities of the glioma cells were detected using a dual-luciferase reporter gene detection kit (Promega E1910) by Promega GloMax 20/20 Luminescence Detector (USA).

### Statistical Analysis

Statistical analysis was performed using SPSS 20.0 (SPSS, Inc. Chicago, USA). Pearson correlation analysis was used in determining correlations between each pair of gene expression levels. A scatter diagram was used for the linear analysis of gene expression. The prognoses of patients with GBM were analyzed using Kaplan–Meier curves. Quantitative values were expressed as (mean ± SD). Each experiment was repeated at least three times. An independent sample *t* test was used for comparison between two groups. *P*<0.05 was considered statistically significant.

## Results

### Identification of the DEGs Between Recurrent and Primary GBM

**Figure 1:**
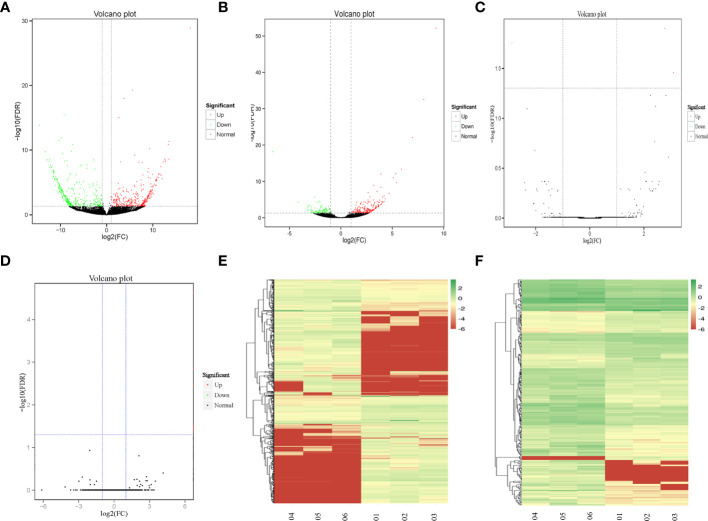
The DEGs between recurrent and primary GBM. **(A–D)** Volcano map of DElncRNAs, DEmRNAs, DEcircRNAs, and DEmiRNAs, expression profiles between recurrent and primary GBMs. The x-axis represents an adjusted a log2FC and the y-axis represents the P-value. The green dots represent down-regulated DEGs, the red dots represent up-regulated DEGs, and the black dots represent non-differentially expressed RNAs. **(E, F)** Unsupervised clustering analysis showing expression profiles of lncRNAs and mRNAs between recurrent and primary GBMs. The color gradient from green to red shows a trend from low expression to high expression.

Through the comparison of the RNA-seq data of three cases of recurrent GBM and three cases of primary GBM, a total of 1025 DEGs were identified, of which 718 were lncRNAs (378 upregulated and 340 downregulated), 293 were mRNAs (204 upregulated and 89 downregulated), 11 were circRNAs (all upregulated), and three were miRNAs (two upregulated and one downregulated). **Figures 1A–D** shows in detail the volcano plots of DElncRNA, DEmRNA, DEcircRNA, and DEmiRNA expression profiles, respectively. Unsupervised clustering analysis shows the expression profiles of lncRNAs and DEmRNAs (**Figures 1E, F**).

### HSPA1A Has Important Biological Functions and Is Upregulated in Recurrent GBM

**Figure 2:**
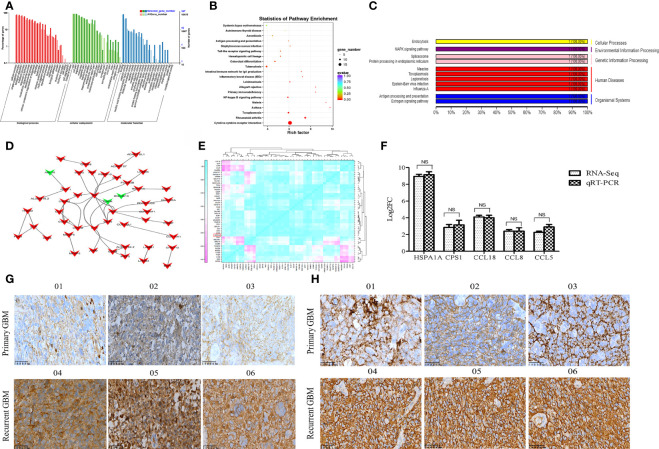
Functional annotation of DEmRNAs and expression validation of selected genes by qRT-PCR and IHC**. (A)** GO classification map of DEmRNAs. **(B)** KEGG functional enrichment map of DEmRNAs. **(C)** KEGG functional classification map of mRNA HSPA1A. **(D)** The PPI map of DEGS mRNA. The DEGs mRNA was indicated by arrows. The up-regulated genes are indicated in red, while the down-regulated genes are indicated in green. **(E)** Correlation analysis of 47 DEmRNAs highly correlated to HSPA1A. **(F)** The differential expression of selected mRNAs was validated by qRT-PCR. **(G)** The IHC image of HSPA1A expression in GBM sample. **(H)** The IHC image of CPS1 expression in GBM specimens. 01-03 represents primary GBM,04-06 represents recurrent GBM.

GO enrichment analysis results showed that the DEmRNAs were markedly enriched in molecular functions, including antioxidant activity, receptor regulator activity, chemoattractant activity, and morphogen activity (**Figure 2A**). The DEmRNAs were significantly enriched in several KEGG signaling pathways, including tumor signaling pathways, immune response, and cytokine and receptor functions (**Figure 2B**). As the highest upregulated DEmRNA in recurrent GBMs (nine times that of primary GBM), HSPA1A, a member of the Hsp70 protein family (Hsp70-1), was mainly related to antigen presentation and MAPK signaling pathway regulation (**Figure 2C**). PPI analysis results showed that carbamoyl-phosphate synthase 1 (CPS1) and Hsp family member 13 (DNAJB3; a co-chaperone and member of the Hsp40 family) are HSPA1A-interacting proteins (**Figure 2D**). A significant correlative relationship was observed between HSPA1A and other proteins, including CPS1 (r=0.996, *P*=0.002), CCL18 (r=0.927, *P*=0.008), CCL8 (r=0.993, *P*<0.001), and CCL5 (r=0.919, *P*=0.010) (**Figure 2E**). Moreover, the expression level of HSPA1A determined by qRT-PCR was consistent with that obtained by RNA-seq (**Figure 2F**). IHC showed that the expression levels of HSPA1A and CPS1 in recurrent GBM were significantly higher than those of primary GBM (**Figures 2G, H**). Therefore, HSPA1A was selected as the target mRNA for screening key lncRNAs.

### NONHSAT079852.2 Targets mRNA HSPA1A and Is Highly Expressed in Recurrent GBM

**Figure 3:**
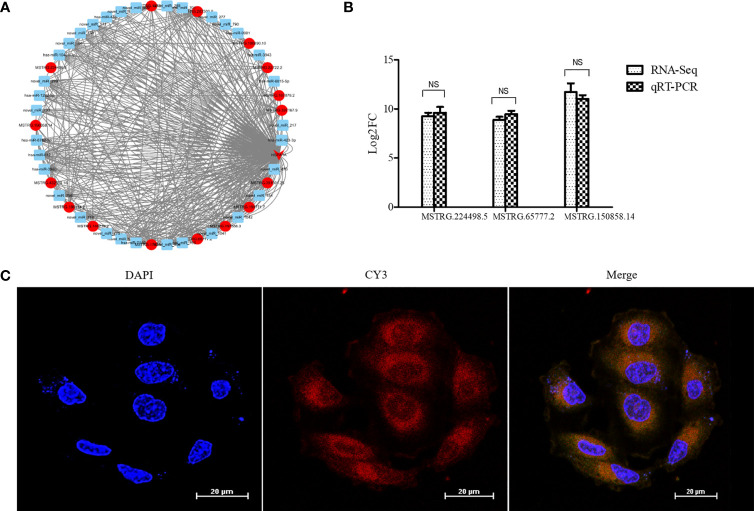
LncRNAs NONHSAT079852.2 targets mRNA HSPA1A and is highly expressed in recurrent GBM and main located in Cytosol. **(A)** The ceRNA network construction of lncRNAs, miRNA and HSPA1A. lncRNAs is indicated by a circle. mRNA is indicated by an arrow. miRNA is indicated by a square. The up-regulated genes are indicated in red, while the genes with insignificant changes are indicated in blue. **(B)** The comparison of qRT-PCR and RNA-seq detection of lncRNAs expression levels in the recurrent GBM and primary GBM. **(C)** Fish experiment demonstrated that the LncRNA NONHSAT079852.2 was mainly distributed in the cytoplasm of glioma cells. The results are presented as mean ± SD. NS, not significant.

According to the results of the analysis of lncRNAs and miRNA target genes, 54 differentially expressed lncRNAs targeted HSPA1A, of which 16 lncRNAs competed with HSPA1A in a ceRNA mode. Meanwhile, 37 DEmiRNAs targeted HSPA1A, and 3761 miRNAs targeted 16 lncRNAs. The 16 lncRNAs, HSPA1A, and their co-targeted miRNAs formed a ceRNA network (**Figure 3A**). The differential expression levels of the 16 lncRNAs were validated by qRT-PCR, and three lncRNAs (MSTRG.224498.5, MSTRG.65777.2, and MSTRG.150858.14) showed results that were consistent with those of RNA-seq (**Figure 3B**). The lncRNA MSTRG224498.5 is also known as lncRNAs NONHSAT079852.2 and belongs to intergenic lncRNA. It is located on chromosome 20 with a length of 1657 bp. Lnclocator shows that lncRNAs NONHSAT079852.2 is located in the cytoplasm (0.113725065505), nucleus (0.0499335493761), ribosome (0.338593997721), cytosol (0.449439437366), and exosome (0.0483079500317) (16). FISH experiment showed that the subcellular location of the NONHSAT079852.2 is the cytosol (**Figure 3C**). Therefore, we selected NONHSAT079852.2 as the key lcnRNA for subsequent analysis.

### NONHSAT079852.2 Can Promote the Proliferation, Invasion, and Migration of Glioma Cells

**Figure 4:**
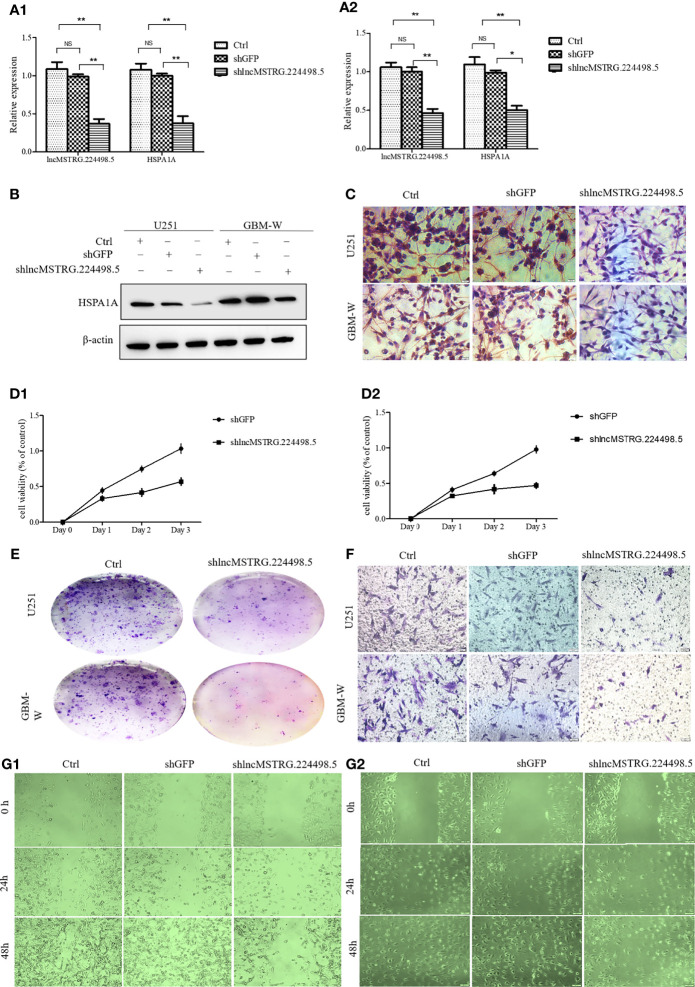
LncRNAs NONHSAT079852.2 can promote the proliferation, invasion and migration of glioma cells. **(A)** qRT-PCR analysis of HSPA1A in U251(A1) or GBM-W(A2) cells after transfection for 48 hours. **(B)** Western blot analysis of HSPA1A in U251 or GBM-W cells after transfection for 48 hours. **(C)** IHC analysis of HSPA1A in U251 or GBM-W cells after transfection for 48 hours. **(D)** Growth curve of U251(D1) or GBM-W (D2) cells after transfection for 48 hours by CCK8 assay. **(E)** Proliferation of U251 or GBM-W cells after transfection for two weeks as determined by colony-formation assay. **(F)** Migration and invasion ability of U251 or GBM-W cells after transfection for 48 hours. **(G)** Migration of U251(G1) or GBM-W(G2) cells after transfection for 48 hours as detected by wound healing assay. Results were presented as mean ± SD. *p < 0.05, **p < 0.01, NS, not significant.

When the lncRNA MSTRG224498.5was knocked down by shRNA, the mRNA and protein expression levels of HSPA1A were reduced (**Figures 4A–C**). CCK-8 assay (**Figure 4 D1, D2**), colony-formation assay (**Figure 4E**), cell migration and invasion assay (**Figure 4F**), and wound healing assay (**Figure 4G**) showed that cell proliferation, invasion, and migration were inhibited.

### NONHSAT079852.2 Can Modulate the Cell Cycle and Apoptosis of Glioma Cells

**Figure 5:**
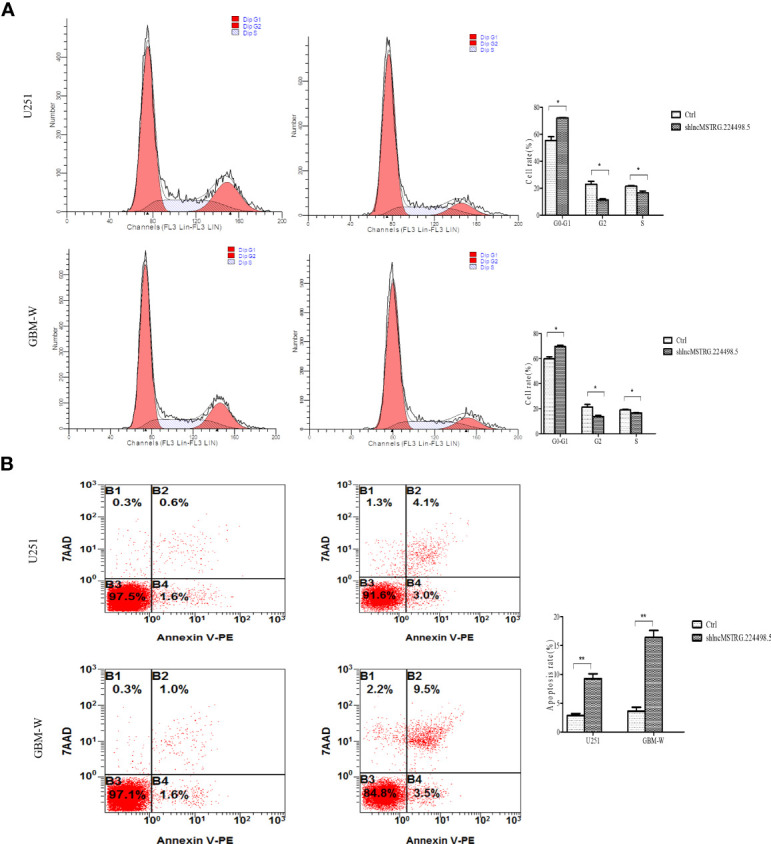
LncRNAs MSTRG224498.5 can adjust the cell cycle and apoptosis of glioma cells. **(A)** Flow cytometry analysis of the cell cycle of U251 or GBM-W cells after transfection for 48 hours. **(B)** Flow cytometry analysis of the apoptosis rate of U251 or GBM-W cells after transfection for 48 hours. Results were presented as mean ± SD. *p < 0.05, **p < 0.01.

When NONHSAT079852.2 was knocked down by shRNA, flow cytometry assay showed that the number of glioma cells in the G_1_/G_0_ phase increased, whereas the number of glioma cells in the G_2_/S phase decreased (**Figure 5A**). In addition, the apoptosis rate of glioma cancer cells increased (**Figure 5B**).

### NONHSAT079852.2 Functions as a ceRNA for has-mir-10401-3p to Facilitate HSPA1A Expression

**Figure 6:**
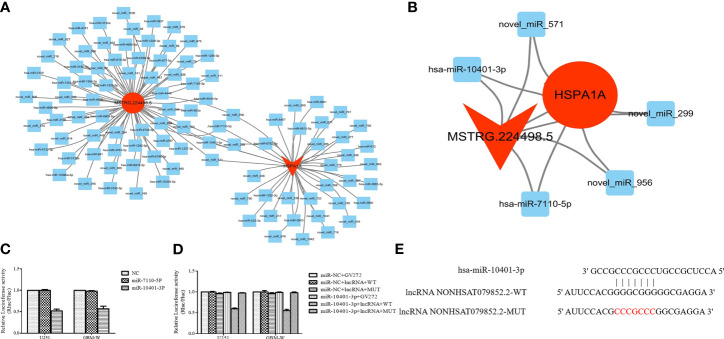
LncRNAs NONHSAT079852.2 Functions as a ceRNA for has-mir-10401-3p to facilitate HSPA1A Expression. **(A)** miRNA targeting HSPA1A and lncRNA NONHSAT079852.2, respectively. **(B)** Co-targeting miRNAs of HSPA1A- lncRNA NONHSAT079852.2; LncRNAs is represented by a circle, miRNA is represented by a diamond, mRNA is represented by an arrow, The up-regulated expression gene is indicated by red, and the gene without significant change is indicated by blue. **(C)** Relative luciferase activity in U251 and GBM-W cells co-transfected with lncRNA MSTRG224498.5 reporter plasmid and candidate miRNAs. **(D)** Relative luciferase activity in glioma cells transfected with wild-type lncRNA NONHSAT079852.2 vector, mutant-type vector or empty vector. **(E)** has-mir-10401-3p and lncRNA NONHSAT079852.2 binding sequences and lncRNA NONHSAT079852.2 mutation sequences.

CeRNA network analysis showed that five miRNAs co-targeted lncRNA MSTRG224498.5 and HSPA1A, and two of them are known miRNAs (**Figures 6A, B**). The RegRNA2.0 database (http://regrna2.mbc.nctu.edu.tw/) showed that NONHSAT079852.2 can interact with miR_571 (novel), hsa-miR-7110-5p, miR_299(novel), miR_956 (novel), and hsa-miR-10401-3p through complementary base pairing. Therefore, NONHSAT079852.2 was speculated to regulate the function of glioma cells by acting as a ceRNA for these miRNAs. The binding ability of NONHSAT079852.2 for the two known miRNAs (hsa-miR-7110-5p and hsa-miR-10401-3p) was further confirmed in glioma cells through an immunofluorescence reporter assay, and the results showed that luciferase activity decreased in the glioma cells that were co-transfected with has-mir-10401-3p and NONHSAT079852.2 but was not reduced in cells containing hsa-miR-7110-5p, Therefore, has-mir-10401-3p was used as a candidate miRNA (**Figure 6C**). We constructed the fluorescent reporter enzyme plasmids NONHSAT079852.2-WT and NONHSAT079852.2-MUT, which contained has-mir-10401-3p binding sites. The upregulation of has-mir-10401-3p significantly reduced luciferase activity in glioma cells co-transfected with NONHSAT079852.2-WT, whereas the upregulation of has-mir-10401-3p had no effect on luciferase activity when the cells were co-transfected with NONHSAT079852.2-MUT. These results suggested that NONHSAT079852.2 bound directly to has-mir-10401-3p (**Figure 6D**). MiRanda and TargetsCAN were used in predicting the possible binding sites of miRNA target genes and regulatory networks, and the results showed the binding sites of has-mir-10401-3p that were co-targeted by lncRNA MSTRG224498.5 and mRNA HSPA1A (**Figure 6E**).

### High NONHSAT079852.2 Expression Is Associated With the Poor Prognoses of Patients With GBM

**Figure 7:**
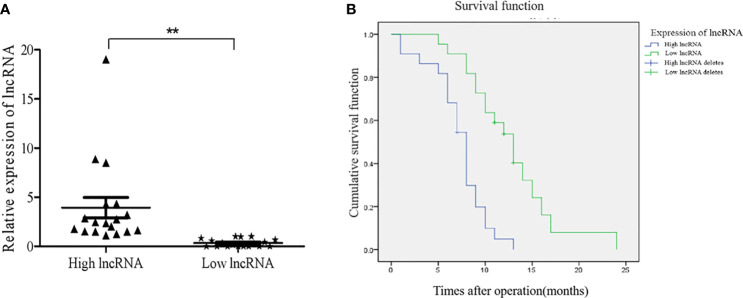
The expression levels of lncRNAs NONHSAT079852.2 in GBM patients and its relationship with the prognosis of patients. **(A)** qRT-PCR analysis of lncRNA MSTRG224498.5 of GBM tissues. **(B)** High expression of lncRNA NONHSAT079852.2 correlates with poor prognosis in GBM patients. Results are presented as mean ± SD. **p < 0.01.

Forty-four patients with primary GBM (19 females and 25 males) who underwent surgery were followed up for 1-24 months. The expression levels of NONHSAT079852.2 in the tumor tissues of 44 patients with GBM were detected through qRT-PCR, and the patients were divided into high- and the low-expression groups according to the median expression level (**Figure 7A**). Kaplan–Meier curves showed that the overall survival time of the high-expression group was significantly lower than that of the low-expression group (Log Rank P=0.000; **Figure 7B**).

## Discussion

To explore the role of lncRNAs in glioma recurrence, we detected the differential transcriptome expression profiles of the recurrent GBM tissues through RNA-seq. After performing lncRNA–mRNA–miRNA ceRNA network analysis, we selected NONHSAT079852.2, which was upregulated in the recurrent GBMs and associated with the poor prognoses of patients with GBM. Through comprehensive bioinformatic analysis and *in vitro* function assays, we found that NONHSAT079852.2 acts as a sponge for has-mir-10401-3p to increase HSPA1A expression and thereby promotes the proliferation, migration, and invasion of glioma cells. Thus, it may be involved in the progression and recurrence of GBM.

Although the functions of most lncRNAs remain unclear, lncRNAs play an important role in tumor initiation and progression by regulating gene expression through diverse mechanisms (17–19). Therefore, through the identification of DEGs and gene functional enrichment analysis, the target mRNA selected was found to be an important factor in tumor pathogenesis. Through mRNA–lncRNA network analysis, we selected interacted lncRNAs that play key roles in recurrent GBM. Using this strategy, we selected HSPA1A as the target mRNA. The major stress-inducible protein HSPA1A is a highly conserved protein of the heat-shock protein 70 (Hsp70) family and plays an important role in protein folding, signal transduction, and general response to stress factors (20–23). Strong evidence suggests that HSPA1A is overexpressed in various tumors, such as lung cancer, gastric cancer, and GBM, and promotes tumor proliferation, metastasis, and drug resistance (24–26). The correlation between increase in Hsp70 expression level and tumor development and progression has prompted scientists to consider Hsp70 as a target for cancer therapy (27–29). Recently, targeting Hsp70 in glioma cells with magnetic nanoparticles has been found to increase the retention of nanoparticles within tumor cells (30, 31). In our study, we found that HSPA1A interacts with CPS1 and DNAJB13, and its expression level was highly correlated with the expression levels of CPS1, CCL18, CCL8, and CCL5. CPS1, a key enzyme in the urea cycle, is highly expressed in different types of cancers and promotes cell proliferation and metastasis (32–34). DNAJB13, one of the HSP40 subfamily members, has a negative correlation with HSPA1A. Hsp40s are cofactors of HSP70s and are involved in various biological processes. The DNAJB1–Hsp70 complex is a potential valuable target for tumor treatment (35, 36). As chemokines, CCL8 and CCL5 are involved in tumor cell proliferation and metastasis (37–39). Basing on the description above, we selected HSPA1A as the target gene for the identification of lncRNAs associated with GBM recurrence.

The lncRNA–mRNA regulatory network showed that HSPA1A, a key gene in the development of GBM, is the targeting gene of NONHSAT079852.2 (lncRNA MSTRG224498.5). The expression of NONHSAT079852.2 significantly increased in recurrent GBM relative to that in primary GBM. Moreover, the knocking down of NONHSAT079852.2 inhibited the proliferation of glioma cells by downregulating HSPA1A expression. These results indicated that NONHSAT079852.2 contributes to GBM recurrence by upregulatimg HSPA1A. NONHSAT079852.2 belongs to intergenic lncRNA and is located on chromosome 20 with a length of 1657 bp. FISH experiment revealed that NONHSAT079852.2 was mainly distributed in the cytoplasm of glioma cells. Therefore, NONHSAT079852.2 might regulate the function of glioma cells through a ceRNA mechanism. Using bioinformatics and luciferase reporter gene assay, we confirmed that NONHSAT079852.2 competitively bound to hsa-miR-10401-3p and thus upregulated HSPA1A expression. In addition, we showed that the high expression of lncRNA MSTRG224498.5 in GBM was associated with poor prognosis. Therefore, MSTRG224498.5 might serve as a putative prognosis marker for patients with recurrent GBM.

This is the first study to characterize the role of the NONHSAT079852.2 in recurrent GBM. GBM clinical specimens were used for RNA-seq analysis. The heterogeneity of these specimens is strong, and this feature provides a reliable foundation for exploring GBM recurrence and drug resistance (40, 41). We established a GBM primary cell line, which maintained the heterogeneity of tumor cells and thus ensured the reliability of the research results (42, 43). In our next study, by using a larger sample size and performing *in vivo* experiments, we will conduct in-depth research and provide reliable evidence for clinical practice.

In summary, our study demonstrated that the NONHSAT079852.2 enhances recurrence and promotes the proliferation, invasion, and migration of glioma cells and these features are related to the poor prognoses of patients with GBM. The tumor-promoting effect of NONHSAT079852.2 may be attributed to its competitive binding to hsa-mir-10401-3p, and the resulting bond upregulates HSPA1A expression. Thus, the NONHSAT079852.2/hsa–mir-10401-3p–HSPA1A axis may be one of the potential mechanisms that promote GBM recurrence and is a potential therapeutic target for controlling and treating GBM.

## Data Availability Statement

The datasets presented in this study can be found in online repositories. The names of the repository/repositories and accession number(s) can be found in the article/**Supplementary Material**.

## Ethics Statement

The studies involving human participants were reviewed and approved by Tangdu Ethics Committee Number TDLL-2017-172. The patients/participants provided their written informed consent to participate in this study.

## Author Contributions

NZ and JZ conducted all experiments and analyzed the data. LW, MC, HC, YJ and YH provided clinical samples. HW, CC, LW and NZ contributed to the conception and design of the study. LZ, XF, QZ collected clinical data. NZ and JZ wrote the manuscript. All authors contributed to the article and approved the submitted version. 

## Funding

This work was supported by the National Natural Science Foundation of China (Grant No. 81702483 and 81772661) and Natural Science Basic Research Program of Shaanxi (Program No. 2019JQ-207 and 2020JZ-30).

## Conflict of Interest

The authors declare that the research was conducted in the absence of any commercial or financial relationships that could be construed as a potential conflict of interest.
