# Corrigendum: Long Noncoding RNA NONHSAT079852.2 Contributes to GBM Recurrence by Functioning as a ceRNA for has-mir-10401-3p to Facilitate HSPA1A Upregulation

**DOI:** 10.3389/fonc.2021.807421

**Published:** 2021-12-07

**Authors:** Ningning Zhao, Jiajie Zhang, Lili Zhao, Xiaoni Fu, Qian Zhao, Min Chao, Haiyan Cao, Yang Jiao, Yaqin Hu, Chao Chen, Liang Wang, Huijuan Wang

**Affiliations:** ^1^ College of Life Sciences, Northwest University, Xian, China; ^2^ Department of Neurosurgery, Tangdu Hospital of Air Force Medical University, Xian, China

**Keywords:** recurrent glioblastoma multiforme, RNA-sequencing, lncRNAs, HSPA1A, ceRNA

In the original article, there were mistakes in **Figure 4** as published. Due to carelessness, we put the wrong picture of **Figure 4B**, we initially planned to use the results of the third repeated experiment as a representative picture, but in the end we put the picture of the first experiment here. The picture for “shGFP” group in **Figure 4C** and **F** were duplication of the images for “Ctrl” group. We had double checked the original data and found that the errors were caused by our carelessness in exporting the representative images and compiling these figures. The corrected **Figure 4** appears below.

**Figure 4:**
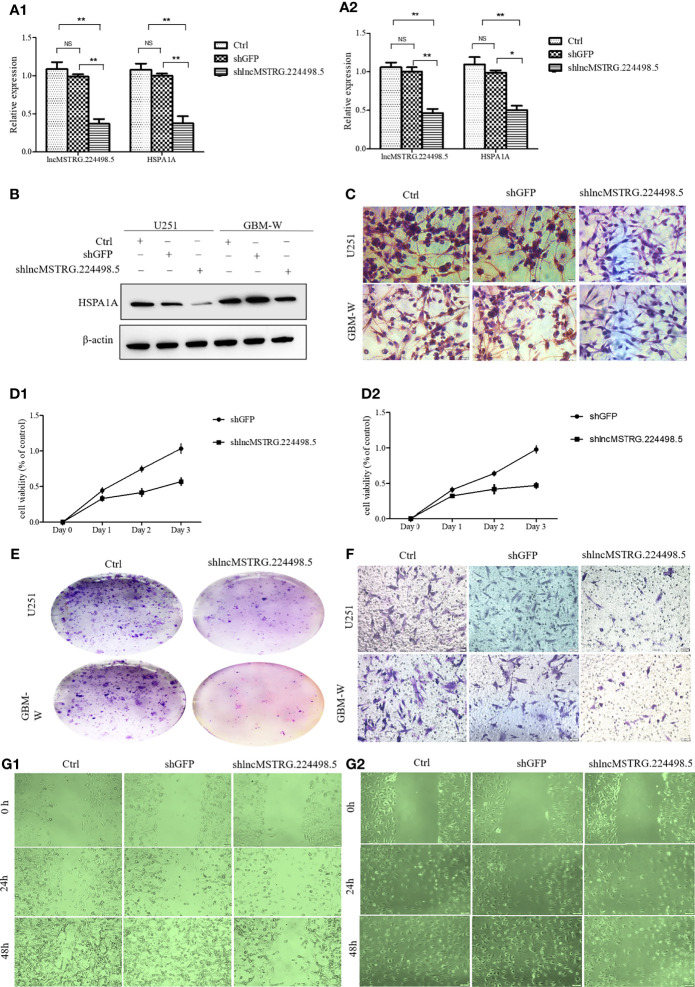
LncRNAs NONHSAT079852.2 can promote the proliferation, invasion and migration of glioma cells. **(A)** qRT-PCR analysis of HSPA1A in U251(A1) or GBM-W(A2) cells after transfection for 48 hours. **(B)** Western blot analysis of HSPA1A in U251 or GBM-W cells after transfection for 48 hours. **(C)** IHC analysis of HSPA1A in U251 or GBM-W cells after transfection for 48 hours. **(D)** Growth curve of U251(D1) or GBM-W (D2) cells after transfection for 48 hours by CCK8 assay. **(E)** Proliferation of U251 or GBM-W cells after transfection for two weeks as determined by colony-formation assay. **(F)** Migration and invasion ability of U251 or GBM-W cells after transfection for 48 hours. **(G)** Migration of U251(G1) or GBM-W(G2) cells after transfection for 48 hours as detected by wound healing assay. Results were presented as mean ± SD. *p < 0.05, **p < 0.01, NS, not significant.

The authors apologize for this error and state that this does not change the scientific conclusions of the article in any way. The original article has been updated.

## Publisher’s Note

All claims expressed in this article are solely those of the authors and do not necessarily represent those of their affiliated organizations, or those of the publisher, the editors and the reviewers. Any product that may be evaluated in this article, or claim that may be made by its manufacturer, is not guaranteed or endorsed by the publisher.

