# Supplementary material for: Long Noncoding RNA NONHSAT079852.2 Contributes to GBM Recurrence by Functioning as a ceRNA for has-mir-10401-3p to Facilitate HSPA1A Upregulation
Source: Front Oncol. 2021 Jul 8;11:636632. doi: 10.3389/fonc.2021.636632 (PMC8297974; doi:10.3389/fonc.2021.636632)
Supplement: Supplementary file 1 [file DataSheet_1.doc]

**Supplementary materials**


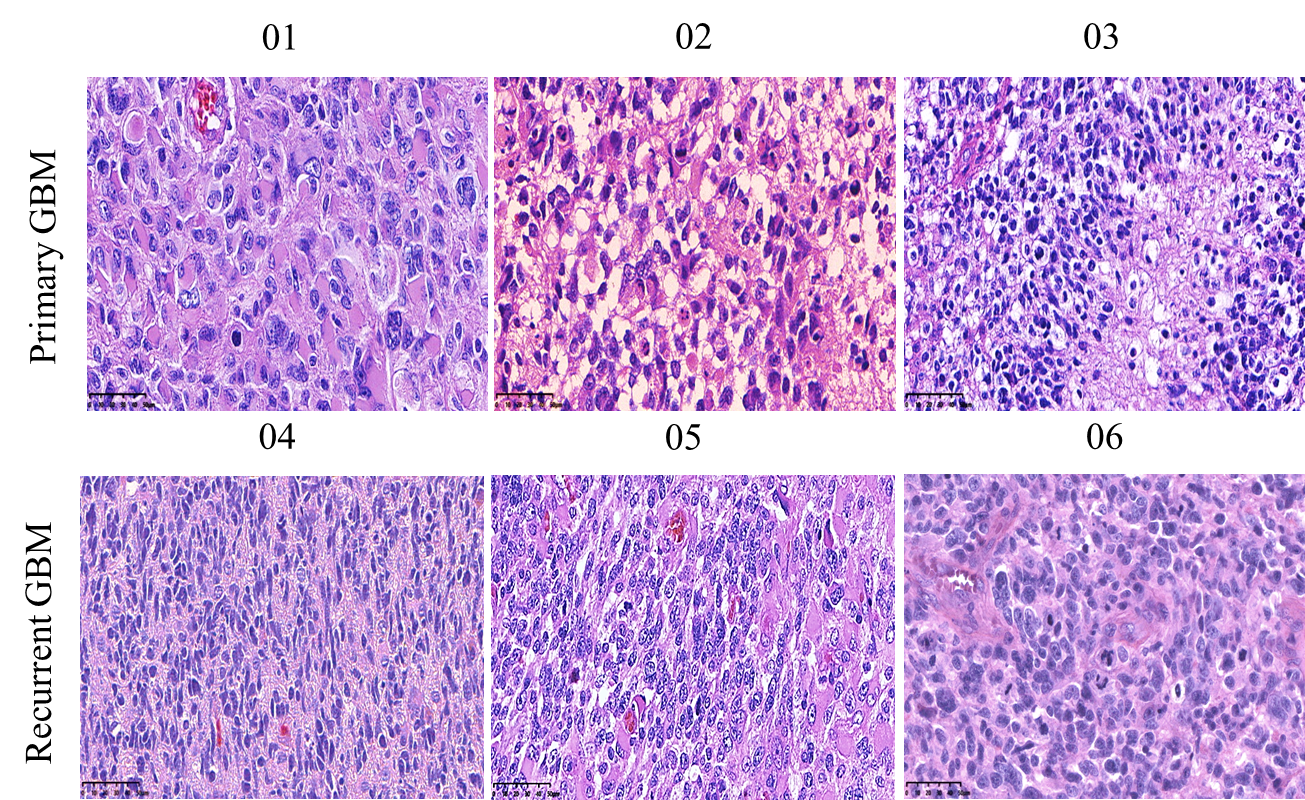


**Supplementary Figure 1** The pathological characteristics of recurrent and primary GBM H&E staining showed that the pathological diagnosis of 3 recurrent GBM and 3 primary GBM were all GBM.


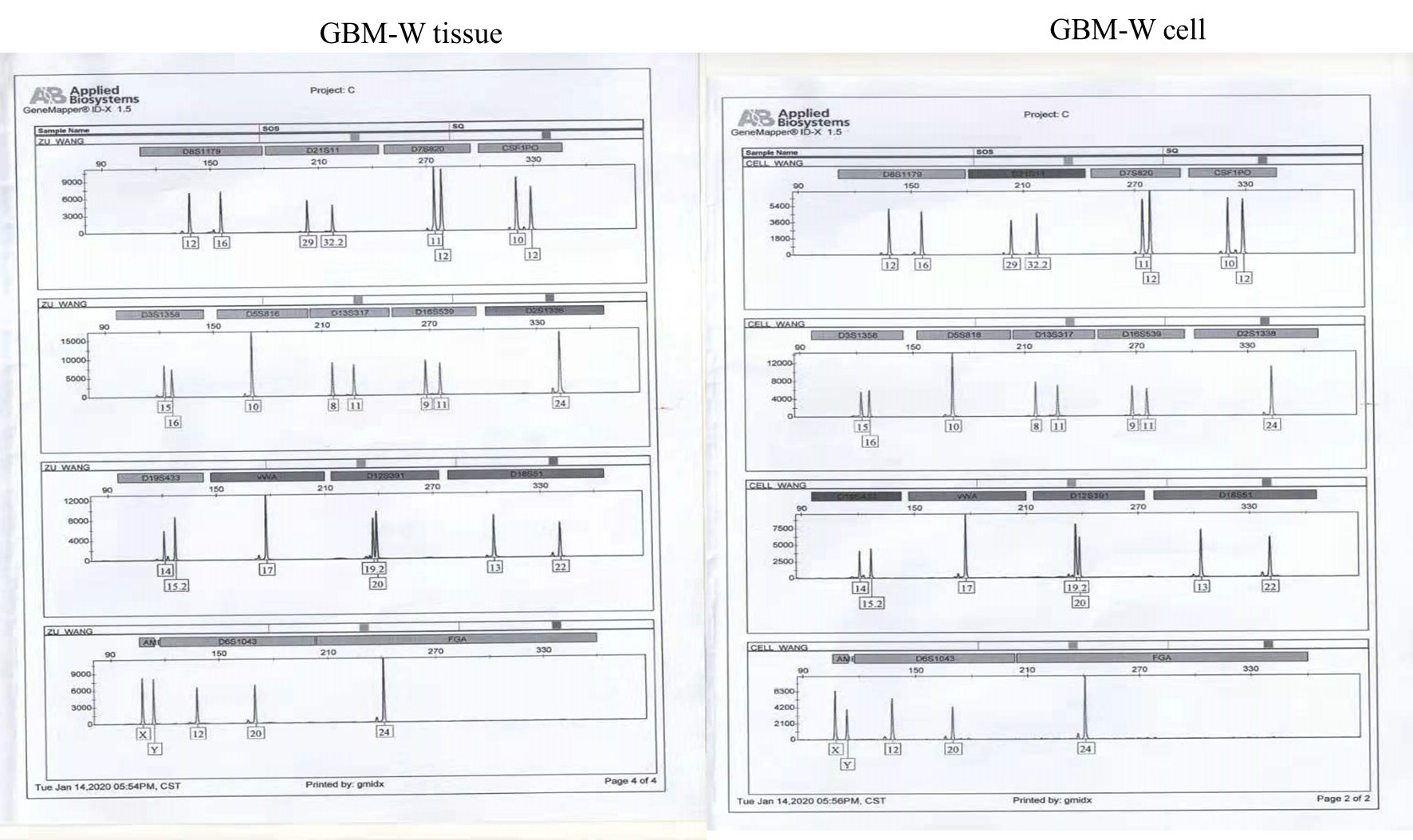


**Supplementary Figure 2** GBM-W, a primary GBM cell line derived from a clinical GBM specimen was obtained from Tangdu Hospital and identified by short tandem repeat (STR) analysis

**Supplementary Table 1 The shRNA targeting sequences for NONHSAT079852.2**

| ID |  |
| --- | --- |
| shRNA-652 | AGGGTTGCATGTTTGGCCCTT |
| shRNA-1319 | GACCATGTGTACTCAATGTTT |
| shRNA-1078 | GCCCTTGTAAGATGAAACAAG |

**Supplemental Table 2** The primer sequences

| Gene | primer | Sequence(5'-3') | Amplification length |
| --- | --- | --- | --- |
| MSTRG.224498.5 | F  R | CACGTGCGCCTTACGTAATTT  ATGACGCCTACTCACTCACC | 129 |
| MSTRG.65777.2 | F  R | CCATCGCCATCGCCTTCCT  ATAACATTCCTGCCCCGTCG | 76 |
| MSTRG .150858.14 | F  R | GGCTTATGTTTGGGCATTTGA  GTCAAGGGACGGAAGAGATCAT | 89 |
| HSPA1A | F  R | AAGAACGCCCTGGAGTCCTACG  CTTGTCCGCCTCGCTGATCTTG | 87 |
| CPS1 | F  R | CAAACAGGCTTTCATTACTGCT  CTCTCATGCATAATCCCCTCAT | 123 |
| CCL18 | F  R | CCTGCCTCAGCGTCCAAAGAAG  GAAGCCAAGAGTGTGAGACCAACC | 120 |
| CCL8 | F  R | CAGTTTCCATTCCAATCACCTG  TTGGTGATTCTTGTGTAGCTCT | 88 |
| CCL5 | F  R | CCAAGTGTGTGCCAACCCAGAG  AGGACAAGAGCAAGCAGAAACAGG | 128 |

**Supplementary Table 3** The Fish probe sequences

| Gene | Sequence(5'-3') |
| --- | --- |
| MSTRG.224498.5 | AGAAAGAGCAGGGCTTGACATCTGACTG |
|  | GGCAGCACGCTTTAGAAAGAGCAGGG |
|  | ACCACTTATCGGGCACTATGCTCACCAC |
